# Dual Targeting of Antioxidant and Metabolic Enzymes to the Mitochondrion and the Apicoplast of Toxoplasma gondii

**DOI:** 10.1371/journal.ppat.0030115

**Published:** 2007-08-31

**Authors:** Paco Pino, Bernardo Javier Foth, Lai-Yu Kwok, Lilach Sheiner, Rebecca Schepers, Thierry Soldati, Dominique Soldati-Favre

**Affiliations:** 1 Department of Microbiology and Molecular Medicine, Centre Medical Universitaire, University of Geneva, Geneva, Switzerland; 2 Department of Biological Sciences, Imperial College London, London, United Kingdom; 3 Department of Biochemistry, Sciences II, University of Geneva, Geneva, Switzerland; Washington University School of Medicine, United States of America

## Abstract

Toxoplasma gondii is an aerobic protozoan parasite that possesses mitochondrial antioxidant enzymes to safely dispose of oxygen radicals generated by cellular respiration and metabolism. As with most Apicomplexans, it also harbors a chloroplast-like organelle, the apicoplast, which hosts various biosynthetic pathways and requires antioxidant protection. Most apicoplast-resident proteins are encoded in the nuclear genome and are targeted to the organelle via a bipartite N-terminal targeting sequence. We show here that two antioxidant enzymes—a superoxide dismutase (TgSOD2) and a thioredoxin-dependent peroxidase (TgTPX1/2)—and an aconitase are dually targeted to both the apicoplast and the mitochondrion of T. gondii. In the case of TgSOD2, our results indicate that a single gene product is bimodally targeted due to an inconspicuous variation within the putative signal peptide of the organellar protein, which significantly alters its subcellular localization. Dual organellar targeting of proteins might occur frequently in Apicomplexans to serve important biological functions such as antioxidant protection and carbon metabolism.

## Author Summary

Toxoplasma gondii is a human and animal pathogen representative of the large group of Apicomplexa. Most members of this phylum contain, in addition to a tubular mitochondrion, a second endosymbiotic organelle indispensable for parasite survival, called the apicoplast. This non-photosynthetic plastid is the site of several anabolic pathways, including the biosynthesis of fatty acids, isoprenoids, iron-sulphur cluster, and heme. Virtually all enzymes active inside the apicoplast are encoded by the nuclear genome and targeted to the organelle via the endoplasmic reticulum courtesy of a bipartite amino terminal recognition sequence. The metabolic activities of the apicoplast impose a high demand for antioxidant protection. We show here that T. gondii possesses a superoxide dismutase and a peroxidase that are shared between the two organelles by an unusual mechanism of bimodal targeting whereby the nature of the signal peptide influences the destination of the protein to both organelles. Dual targeting also extends to other classical metabolic enzymes such as aconitase, uncovering unexpected metabolic pathways occurring in these organelles. In consequence, the bioinformatic predictions for plastidic or mitochondrial targeting on the basis of the characteristics of N-terminal presequences are insufficient in the absence of an experimental confirmation.

## Introduction

### Plastids and Mitochondria in Apicomplexans

The phylum Apicomplexa comprises important obligate intracellular parasites, including Plasmodium falciparum (Pf), the causative agent of the most deadly form of malaria, and *Toxoplasma,* which is responsible for toxoplasmosis in humans and animals. Most Apicomplexans possess a relic plastid organelle called apicoplast, the result of the ancient secondary endosymbiotic uptake of a red algal–like eukaryote. This organelle is non-photosynthetic but fulfils a number of functions that are critical for parasite survival and thus confer sensitivity to antibiotics. Notably, this compartment is the site of biosynthesis of isoprenoids, fatty acid (type II), iron-sulphur cluster, lipoic acid, and part of the heme, therefore rendering it a promising target for novel anti-malarial therapies [1–5]. Additionally, reducing power in the form of ferredoxin is probably produced within the apicoplast by ferredoxin-NADPH reductase (FNR) [6]. The vast majority of apicoplast proteins are nuclear encoded and targeted to the organelle via a bipartite, N-terminal sequence extension composed of a signal peptide (SP) targeting the nascent polypeptide to the endoplasmic reticulum (ER), followed by a transit peptide rich in basic amino acids that acts like the transit peptides targeting to mitochondria and chloroplasts [7–9]. Most Apicomplexans possess a single tubular mitochondrion that contributes to heme biosynthesis and hosts enzymes involved in iron-sulphur cluster synthesis, Krebs's cycle (tricarboxylic acid [TCA] cycle), and in oxidative respiration [10,11]. Biochemical evidence confirms that respiration and oxidative phosphorylation occur in the mitochondria of T. gondii (Tg) and *Plasmodium* [12,13]. However, the physiological relevance of the TCA cycle and respiration may vary throughout the various life stages of *Plasmodium,* and very little is known about T. gondii in this regard.

### Oxidative Stress and Antioxidant Defense in T. gondii

All aerobic organisms require mechanisms that limit molecular damage caused by reactive oxygen species (ROS) and iron stress. ROS, including hydrogen peroxide, superoxide, and hydroxyl radicals, are generated by the incomplete reduction of oxygen during respiration in mitochondria and as side products of a variety of metabolic reactions in many cellular compartments. Excess intracellular iron may aggravate ROS formation and lead to the generation of highly damaging ferryl ions and oxygen-bridged Fe(II)/Fe(III) complexes. Furthermore, intracellular pathogens like T. gondii must protect themselves against the oxidative burst imposed by the host. Previous studies revealed the existence of an antioxidant network in T. gondii [14–17] that includes one cytosolic and two mitochondrial superoxide dismutases (SODs) that catalyze the first step in the enzymatic detoxification of oxygen radicals by converting O_2_^•−^ into molecular oxygen and hydrogen peroxide (H_2_O_2_). A cytosolic catalase, two cytosolic peroxiredoxins (TgPRX1 and TgPRX2), and a mitochondrial peroxiredoxin (TgPRX3) have recently been identified and proposed—together with TgTPX1, a putative thioredoxin-dependent peroxidase (TPX)—to act downstream of the SODs to detoxify hydrogen peroxide [17–19]. Despite the presence of metabolic pathways generating both iron and oxidative stress, to date it has been unclear how the apicoplast protects itself against oxidative damage, because no antioxidant enzyme has been identified in this organelle. TgSOD2 was reported to localize to the parasite mitochondrion despite the presence of a large N-terminal extension that resembles the N-terminal bipartite targeting signals, which typically route proteins to the apicoplast [16,17]. In plants, several antioxidant proteins, such as glutathione reductase and ascorbate peroxidase [20,21] are directed to both the mitochondria and the chloroplast via dual targeting.

### Dual Targeting of Proteins

There are several ways for cells to localize identical or similar proteins in different subcellular compartments [22]. One possibility is through gene duplication during evolution along with acquisition of different targeting signals. Alternatively, multiple products resulting from one single gene locus by differential RNA splicing or by usage of alternative translational initiation sites from the same messenger RNA [23–26] may lead to different protein localizations. Translational initiation from non-AUG codons has also been reported to occasionally contribute to the generation of alternative protein species from the same transcript [27,28]. Another possibility is for one single protein species to be targeted to multiple locations. This can be achieved either by similar molecular receptors and import machineries recognizing similar targeting information—e.g., an N-terminal transit peptide in the case of dual targeting to plastids and mitochondria in plant cells [29]—or by multiple distinct receptors and import machineries recognizing different parts of the protein. We refer to this last case as “bimodal targeting”, and it has previously been documented for the targeting of cytochromes P4502B1 and P4502E1 and of the Slit3 protein to the ER and mitochondria in mammalian cells [30–32], as well as for the chloroplast- and ER-localized protein disulfide isomerase RB60 in the green alga Chlamydomonas reinhardtii [33].

The apparent lack of presumably necessary antioxidant defense enzymes in the apicoplast of T. gondii and P. falciparum and the widespread occurrence of dual organellar targeting of such enzymes and other proteins in other organisms prompted us to revisit the localization of SOD2 in these Apicomplexans. We report here that this protein is dually targeted to both the mitochondrion and the apicoplast of *T. gondii,* most likely in a bimodal fashion. Following this finding, we identified two further enzymes—the putative TPX TgTPX1/2 and the aconitase/iron regulatory protein (IRP)–like protein TgIRP—that are targeted to both the mitochondrion and the apicoplast of *T. gondii,* and we discuss possible metabolic rationale and purpose of the dual organellar localization of these proteins in T. gondii.

## Results

### Apicomplexan SOD2 Exhibits an Unusual Bipartite N-Terminal Targeting Signal

**Figure 1:**
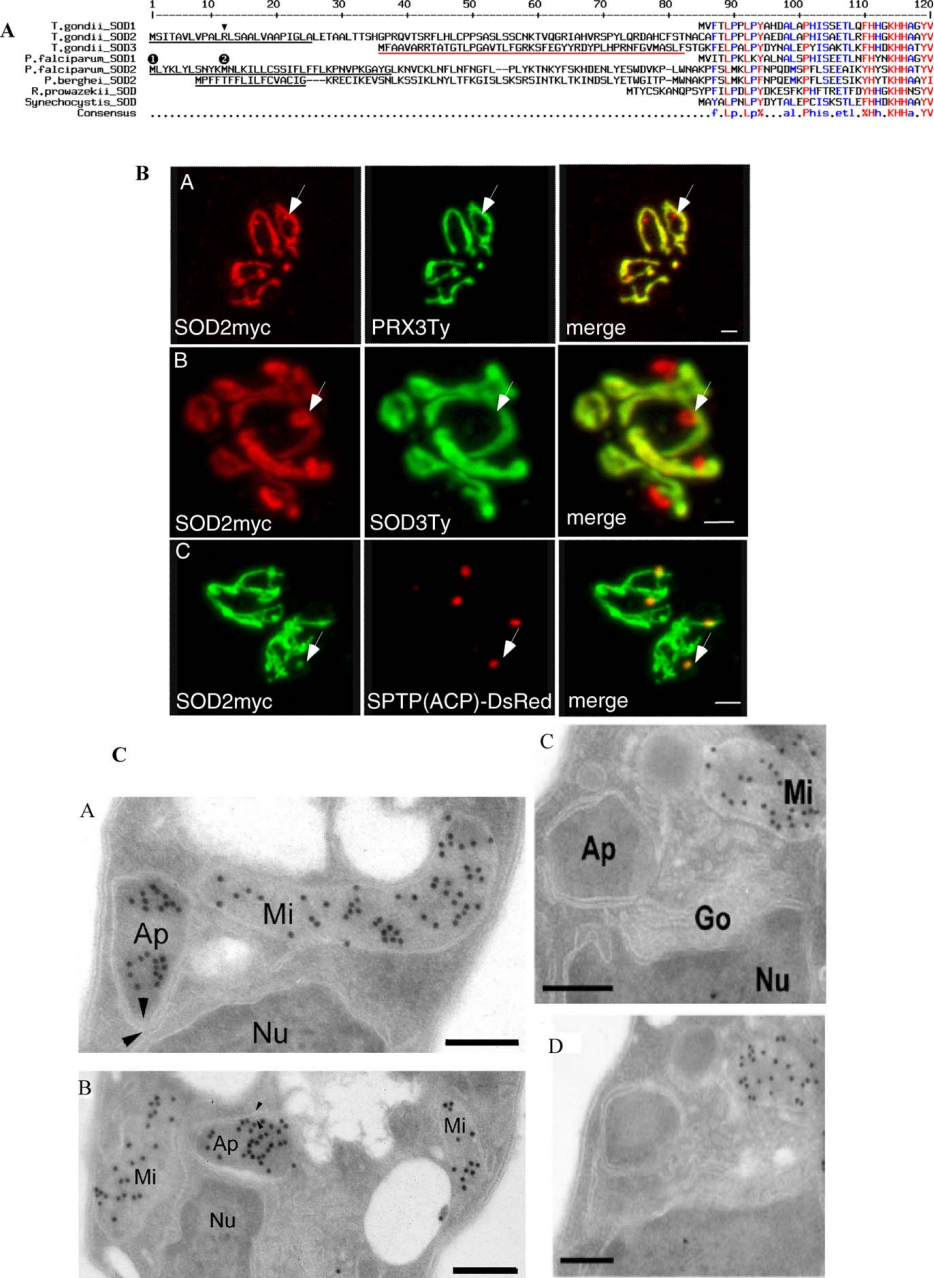
TgSOD2 Contains an Unusual Bipartite N-Terminal Signal and Is Targeted to both Mitochondrion and Apicoplast (A) Alignment of the N-termini of SOD sequences from apicomplexan parasites and two bacteria. Putative SPs as predicted by SignalP are underlined in black, and putative mitochondrial transit peptides as predicted by TargetP and MitoProt are underlined in red. The arrowhead denotes the arginine residue mutated in another study [16]. The numbers in black circles above the PfSOD2 sequence denote the two first methionines. (B) Bimodal targeting of TgSOD2. Incomplete colocalization of SOD2myc with the two mitochondrial markers PRX3Ty and SOD3Ty shows that SOD2 is targeted to both the mitochondrion and an additional compartment (panels A and B). Colocalization of SOD2myc (in green) with the apicoplast marker SPTP(ACP)-DsRed demonstrates that SOD2 is also targeted to the apicoplast (panel C). Scale bars represent 2 μm. (C) Immunoelectron microscopy on parasites stably expressing tagged proteins reveals SOD2Ty to be present in both the mitochondrion and the apicoplast of T. gondii (panels A and B), whereas SOD3Ty exclusively localizes to the parasite mitochondrion (panels C and D). Panels C and D depict two serial sections of the same cell (one section in between not shown). Ap, apicoplast; Go, Golgi apparatus; Mi, mitochondrion; Nu, nucleus. The arrowheads indicate the multiple membranes surrounding the apicoplast. Scale bars represent 200 nm.

Three SODs have been described in *T. gondii:* TgSOD1 is cytoplasmic [15], whereas TgSOD2 and TgSOD3 (not expressed in tachyzoites) have been reported to be mitochondrial based on epitope tagging and green fluorescent protein (GFP) fusion experiments [16,17]. Despite its mitochondrial localization, TgSOD2 exhibits an N-terminal extension that consists of a putative SP (underlined in Figure 1A) followed by a hydrophilic section with a net positive charge, thus showing the essential characteristics of bipartite N-terminal targeting sequences that commonly direct proteins to the apicoplast [8,34]. In contrast to *T. gondii, Plasmodium* species possess only two *SOD* genes, and in *P. falciparum,* the N-terminus of PfSOD2 has been reported to target GFP to the mitochondrion [35]. We confirmed the N-terminal sequence extension of PbSOD2 by reverse transcription (RT)-PCR amplification from a P. berghei (Pb) cDNA library and show that, like TgSOD2, the N-terminal sequences of both PbSOD2 and of PfSOD2 contain a hydrophobic segment followed by an amphipathic helix (Figure 1A), which is suggestive of a targeting signal to the apicoplast. Considering the apparent lack in the plastid organelle of antioxidant enzymes and of SOD in particular, we reassessed the localization of TgSOD2 and PfSOD2 by generating stable transgenic parasites expressing tagged proteins.

### TgSOD2 is Bimodally Targeted to Both the Mitochondrion and the Apicoplast

C-terminally myc-tagged TgSOD2 (TgSOD2myc) colocalized with two previously identified mitochondrial proteins, TgSOD3Ty and TgPRX3Ty [17] (Figure 1B). But in addition to the mitochondrion, TgSOD2myc also localized to a single closely associated compartment whose morphology and position within the cell was suggestive of the apicoplast. A colocalization of TgSOD2myc with an established apicoplast marker, the SPTP(ACP)-DsRed fusion [7], confirmed the localization of SOD2 to the plastid organelle. The presence of SOD2 in the two organelles was also confirmed by immunoelectron microscopy. Figure 1C shows T. gondii tachyzoites with typical organelle topology: the Golgi is anterior to and in close contact with the nucleus, while the apicoplast, surrounded by multiple membranes, is situated adjacent to the Golgi. TgSOD3 is only localized to the mitochondrion, readily identifiable by its internal cristae. We refer to the process giving rise to this dual localization of SOD2 as “bimodal” targeting because (1) the absence of a second methionine (apart from the start-methionine) in the SOD2 N-terminal extension (amino acids 1–91) rules out that the double organellar localization is caused by alternative translation initiation sites, and because (2) the construct encoding SOD2myc contained the cloned cDNA of this gene, making differential splicing highly unlikely. In addition, in a previous study, Brydges and Carruthers demonstrated that a single mutation of an arginine to an alanine residue within the SP of TgSOD2 optimized this signal and led to a complete translocation of the protein into the secretory pathway and trageting to the apicoplast [16]. This experiment strongly indicates that dual localization is not due to a translational initiation from a non-AUG codon [27,28].

### Information in the Mature SOD Protein Region Is Necessary for Bimodal Targeting

**Figure 2:**
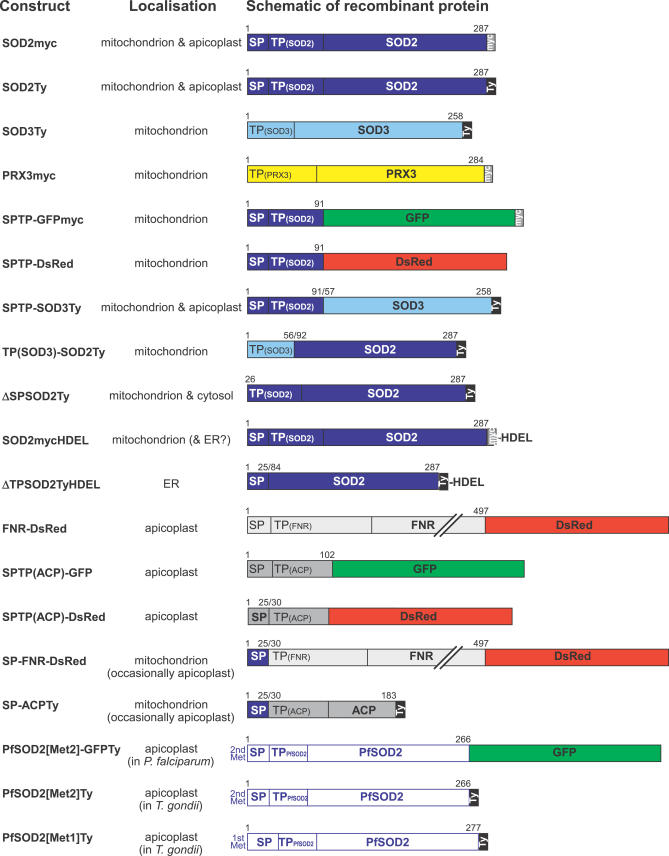
Overview of Recombinant Constructs Expressed in T. gondii Various T. gondii full-length proteins and protein domains were expressed as tagged fusion proteins (employing myc- or Ty-protein tags, DsRed, and/or GFP) to determine their intracellular localization. The overview shows the name of the recombinant construct, the experimentally determined localization, and a diagrammatic representation of the fusion protein indicating the exact boundaries of protein domains (numbers refer to amino acids). If not specified further, SP, TP, and SPTP refer to the predicted signal peptide, transit peptide, and the putative bipartite leader of TgSOD2, respectively. Brackets indicate SP and TP domains taken from other proteins (SOD3 or ACP). For example, SPTP-SOD3Ty refers to the putative bipartite leader of SOD2 (amino acids 1–91; dark blue) followed by the predicted mature protein portion of SOD3 (amino acids 57–258; light blue) followed by a C-terminal Ty-tag, whereas TP(SOD3)-SOD2Ty refers to the predicted mitochondrial transit peptide of SOD3 (amino acids 1–56; light blue) followed by the predicted mature protein portion of SOD2 (amino acids 92–287; light blue) followed by a C-terminal Ty-tag.

**Figure 3:**
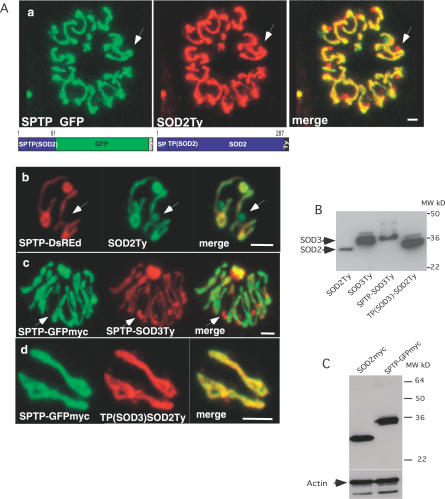
The N-Terminal Targeting Sequence of SOD2 Is Not Sufficient for Bimodal Targeting of Reporter Proteins (A) The predicted bipartite leader of SOD2 (SPTP) fused to reporter proteins (GFPmyc or DsRed) only localizes to the parasite mitochondrion as revealed by colocalization with the bimodally targeted SOD2 (SOD2Ty) (panels a and b), indicating that part of the information necessary for bimodal targeting of SOD2 resides in its mature protein portion. Replacement of the GFP or DsRed reporter by the predicted mature protein of SOD3 complements the bipartite leader of SOD2 (SPTP-SOD3Ty) and restores bimodal targeting to both organelles (panel c). The putative mitochondrial transit peptide of SOD3 in conjunction with the SOD2 mature protein leads to mitochondrial localization (panel d). The arrows indicate the apicoplast. Scale bars represent 2 μm. (B) Western blot analysis of parasites stably expressing SOD2Ty, SOD3Ty, SPTP-SOD3Ty, and TP(SOD3)-SOD2Ty using anti-Ty-1 antibodies. (C) Western blot analysis of parasite clones stably expressing SOD2myc and SPTP(SOD2)-GFPmyc using anti-myc and anti-actin antibodies, showing that the bimodal targeting of SOD2myc is not due to a difference in the expression level compared with the mitochondrion-targeted SPTP-GFPmyc.

To define the sequence information responsible for bimodal targeting of TgSOD2, we generated a number of chimeric constructs (Figure 2). The N-terminal extension of TgSOD2 (amino acids 1–91, SPTP) was fused to two reporter proteins, GFP and DsRed, and stably transfected into T. gondii. Unexpectedly, these fusion proteins (SPTP-GFPmyc and SPTP-DsRed) localized to the mitochondrion, but not to the apicoplast (Figure 3Aa and 3Ab), suggesting that the bipartite N-terminal leader sequence of SOD2 is insufficient for bimodal targeting, or that the artificial junction created by the fusion with the reporter proteins impaired proper targeting. In contrast, fusing the N-terminal extension of SOD2 to the mature protein portion of SOD3 (SPTP-SOD3Ty) resulted in bimodal targeting. This was confirmed by colocalization of SPTP-SOD3Ty with SPTP-GFPmyc (expressed stably and localized in the mitochondrion only; Figure 3Ac) and with the apicoplast marker FNR-DsRed (unpublished data). Notably, SPTP-SOD3Ty appeared to be concentrated more in the apicoplast than in the mitochondrion when compared to SOD2Ty. Reversing this experiment by fusing the mature protein part of SOD2 to the mitochondrial transit peptide of SOD3 (amino acids 1–56) resulted in exclusive mitochondrial localization of the tagged protein TP(SOD3)-SOD2Ty (Figure 3Ad), which demonstrates that the targeting information in the mature SOD2 contributing to apicoplast targeting of SOD2Ty does not impair or override the function of the N-terminal mitochondrial transit peptide of SOD3.

### Processing of TgSOD2

**Figure 4:**
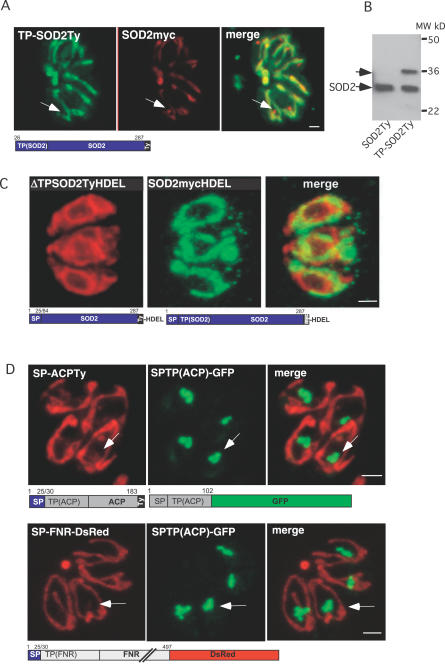
Dissection of the Bipartite Targeting Signal of TgSOD2 (A) Double IFA on parasites expressing stable TP-SOD2Ty and transient SOD2myc showed that removal of the SP abolishes targeting to the apicoplast, localizing the protein to the mitochondrion and the cytosol. The arrows indicate the apicoplast. Scale bars represent 2 μm. (B) Western blot analysis using anti-Ty-1 antibodies confirms incomplete targeting of TP-SOD2Ty to the organelle by showing that a fraction of the protein remains unprocessed. The putative SP of TgSOD2 acts as a suboptimal SP by only partially targeting SOD2 to the apicoplast. (C) Colocalization of parasites expressing SOD2TyHDEL and ΔTPSOD2TyHDEL. Insertion of an ER retention signal on SOD2 that lacks the TP(ΔTPSOD2TyHDEL) leads to accumulation of the protein in the ER while insertion of HDEL on SOD2 (SOD2TyHDEL) does not prevent targeting to the mitochondrion (Figure 4C). This demonstrates that SOD2 is transported independently to the two organelles. (D) When fused to the protein FNR or ACP, the SP of TgSOD2 caused a dramatic relocalization of FNR and ACP to the mitochondrion with occasional apicoplast accumulation. The arrows indicate the apicoplast.

Proteins targeted to mitochondria and plastids are usually proteolytically processed upon import into the organelle. The processing of SOD fusion proteins was investigated by western blot using anti-Ty antibodies and total cell lysates from stably transfected parasites (Figure 3B). The two SOD fusion proteins with dual organellar localization (SOD2Ty and SPTP-SOD3Ty, see Figure 2) were detected as a single band each (Figures 3B and 4B), indicating that the N-terminal leader sequence of SOD2 (SPTP) is cleaved at probably the same site in both organelles. Likewise, the two fusion proteins that are targeted to mitochondrion only (SOD3Ty and TP(SOD3)-SOD2Ty) show one major band each, although faint bands of slightly higher molecular weight are detectable and likely correspond to precursor molecules.

### Both Parts of the Bipartite Leader Sequences Are Necessary; Direct Traffic between the Two Organelles Can Be Excluded

A previous study employing deletion constructs had reported that both the SP-like and the transit peptide–like regions of TgSOD2 are required for correct targeting [16]. Likewise, we found that similar constructs (ΔTPSOD2Ty, Figure 2) yielded fluorescence in the secretory pathway but not in the mitochondrion or the apicoplast (unpublished data). Consistent with this finding, appending an ER retention signal to these constructs resulted in labeling of the ER (construct ΔTPSOD2TyHDEL, Figure 4C; [16]). Second, deletion of the putative SP (ΔSPSOD2Ty, Figure 2) resulted in a partial protein import into the mitochondrion (Figure 4A). This was confirmed by western blot analysis (Figure 4B), which showed that half of the protein was fully processed (presumably upon import into the mitochondrion), whereas the remainder was detected as higher molecular weight form and likely represented unprocessed proprotein in the cytosol.

The mitochondrion and apicoplast organelles are very tightly connected, and attempts to physically separate them by diverse experimental procedures have failed to date [36]. This intimate association could reflect the existence of a direct flow of molecules from one organelle to the other. To exclude direct traffic of SOD2 between the two organelles, we inserted an ER retention signal on SOD2 (SOD2mycHDEL). The HDEL signal interfered only with apicoplast targeting but did not affect mitochondrial targeting (Figure 4C), which suggests that SOD2 is transported independently to the two organelles.

### The Putative SOD2 SP Re-Routes Classical Apicoplast Proteins to the Mitochondrion

A classical N-terminal SP (in proteins bound for the apicoplast elsewhere in the secretory pathway) causes co-translational protein import into the ER, and therefore prevents post-translational import into the mitochondrion. We reasoned that the essential difference between the bipartite N-terminal extension of TgSOD2 and a classical apicoplast targeting signal is due to the suboptimal nature of the putative SOD2 SP. If not all the nascent polypeptides associate with the signal recognition particle and dock to the ER, translation could pursue and uncover a targeting signal to the mitochondrion. Such unusual properties of the putative TgSOD2 SP were demonstrated by replacing the SPs of two established apicoplast-resident proteins (FNR and ACP) with SP of TgSOD2. Immunofluorescence assay (IFA) on stably transfected parasites revealed that both fusion proteins (SP-FNR-DsRed and SP-ACPTy; Figure 2) were targeted predominantly to the mitochondrion and only minor fractions were still localized to the apicoplast (Figure 4D).

### PfSOD2 Localizes to the Apicoplast throughout the Intra-Erythrocytic Cycle

**Figure 5:**
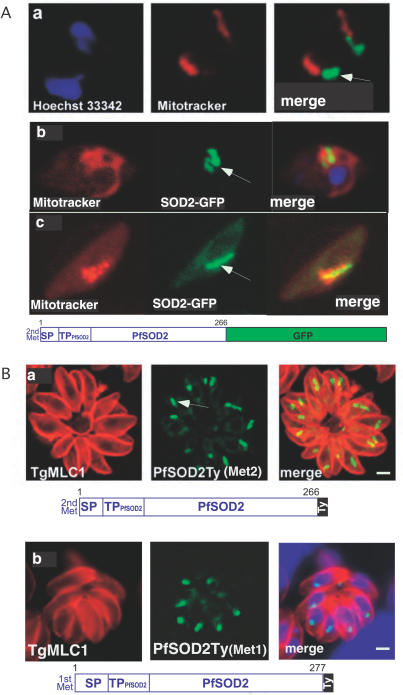
PfSOD2 Is Targeted to the Apicoplast in the Intra-Erythrocytic Stages of P. falciparum (A) IFA on live erytrocytes infected with transgenic P. falciparum parasites expressing PfSOD2GFP. The construct starts either at the second methionine ( MNLKIL…) and is controlled by the *PfCRT* promoter (panel a), or at the first methionine (MLYKLY…) and is controlled by the *PfSOD2* promoter (panel b) (see Figure 1A). The mitochondrion is labeled in red with MitoTracker and the localization PfSOD2-GFP to the apicoplast is detected in green. The same construct was stably expressed in the strain 3D7 and the same results were observed. Moreover, PfSOD2 was also shown to localize to the apicoplast only in the gametocytes (panel c). The arrows indicate the apicoplast. (B) IFA on stable transgenic T. gondii parasites expressing *PfSOD2* with a Ty tag at the C-terminus. The construct starts either at the second methionine (panel a) or at the first methionine (panel b). The periphery of T. gondii parasites is labeled in red with anti-MLC1 antibodies, while the localization of the fusion protein PfSOD2-Ty to the apicoplast is shown in green.

PfSOD2 was previously described as a mitochondrial enzyme based on the ability of its N-terminal leader sequence to target GFP to this organelle [35]. However, the full-length *SOD2* gene from P. falciparum expressed as a tagged protein in either P. falciparum or T. gondii is highly suggestive of apicoplast localization only (Figure 5). Localization of PfSOD2 to the apicoplast was observed in the intra-erythrocytic stages of P. falciparum with PfSOD2-GFP either (1) starting at the “second” Met (as predicted by the PHAT3, and as used in the previous study [35], and controlled by the chloroquine resistance transporter *(CRT)* promoter (Figure 5Aa), (2) or starting at the “first” Met (located 11 amino acids upstream of the “second” Met; Figure 1A) and controlled by the endogenous *SOD2* promoter (Figure 5Ab). Generation of transgenic parasites with this latter construct in the strain 3D7 confirmed that PfSOD2 is also restricted to the apicoplast in the gametocytes (Fig5Ac). Heterologous expression of the PfSOD2 as a Ty-tagged protein in T. gondii corroborates these results (Figure 5Ba and 5Bb) and indicates that the putative apicoplast targeting of PfSOD2 we observe is due neither to the influence of GFP or the Ty-tag, nor to the inclusion of the 11 amino acids preceding the annotated gene sequence (AAT11554). Moreover, although the timing of gene expression throughout the intra-erythrocytic stages is tightly controlled, we have excluded an influence of the promoter by obtaining identical results when using the endogenous *SOD2* promoter (Figure 5Bb). The presence or absence of mature PfSOD2 sequence might play a critical role, as observed for TgSOD2.

### Dual Organellar Targeting of a TPX

**Figure 6:**
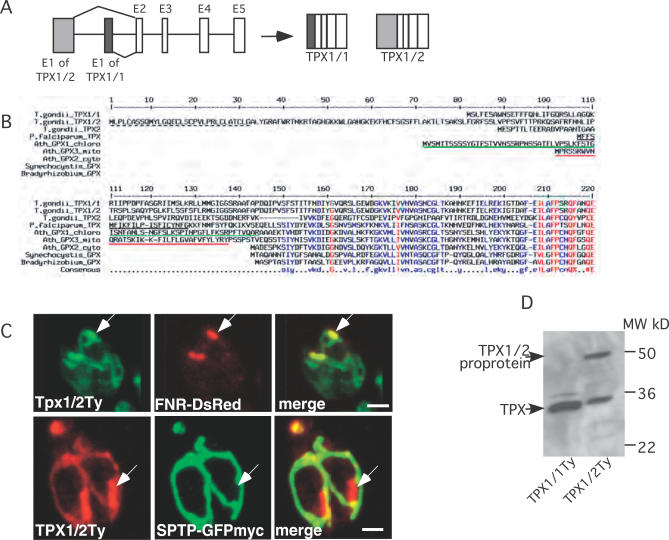
Dual Targeting of a T. gondii TPX (A) Schematics showing the generation of two transcript variants consisting of five exons each (E1–E5) by differential splicing and the resulting gene products TPX1/1 and TPX1/2. (B) Alignment of apicomplexan TPX sequences and related glutathione peroxidases. Putative SPs as predicted by SignalP are underlined in black (a broken line indicates a positive prediction by the Hidden Markov Models only, and not by the Neural Networks), and putative mitochondrial or chloroplast transit peptides as predicted by TargetP or MitoProt are underlined in red or green, respectively. (C) Parasites stably expressing TgTPX1/2Ty are transiently transfected with the apicoplast and mitochondrial markers, FNR-DsRed and SPTP-GFPmyc, respectively. Double IFA reveals dual targeting of TgTPX1/2Ty to both organelles. The arrows indicate the apicoplast. Scale bars represent 2 μm. (D) Western blot analysis (using anti-Ty-1 antibodies) reveals processing of TgTPX1/1Ty and TPX1/2Ty in parasites stably expressing these proteins.

SODs take part in the first step of oxygen radical detoxification that generates hydrogen peroxide, itself a toxic molecule. The presence of a SOD in the apicoplast thus suggested that a complementary peroxiredoxin or peroxidase should also be localized in this organelle to prevent the Fenton's reaction. TgPRX3 was previously described as a mitochondrial peroxiredoxin [17] and was confirmed here to localize exclusively to this organelle (Figure 1B). In contrast, TgPRX1 and TgPRX2, as well as catalase, were reported to be cytosolic [14,17]. Interestingly, a TPX (PfTPX) was previously described in *P. falciparum* [37–39] and expected to be apicoplast-targeted by the neural network tool PATS [40]. A homolog of PfTPX is present in the genome of *T. gondii,* and specific expressed sequence tags revealed two putative mRNAs that differ only in the 5′-ends of the predicted translated region. We experimentally confirmed the existence of these two alternative transcripts by RT-PCR amplification (Figure 6A). The predicted gene products, TgTPX1/1 and TgTPX1/2, correspond to polypeptides of 251 and 333 amino acids length, respectively (Figure 6B). Compared to the similar cytosolic Arabidopsis thaliana glutathione peroxidase AtGPX2, TgTPX1/1 and TgTPX1/2, as well as PfTPX, feature N-terminal extensions suggestive of organellar targeting. Furthermore, as in TgSOD2, the N-terminal extension of TgTPX1/2 resembles a bipartite apicoplast-targeting presequence [7,34]. We determined the subcellular localization of the two TgTPX1 isoforms by IFA in parasites stably expressing the full-length TPX1 proteins C-terminally tagged with Ty. TgTPX1/1Ty was essentially cytoplasmic (unpublished data), whereas TgTPX1/2Ty was like TgSOD2 localized both to the apicoplast and mitochondrion (Figure 6C). Western blot analysis revealed the existence of two protein species (about 50 kDa and 33 kDa) of TgTPX1/2Ty, suggesting N-terminal processing of this protein (Figure 6D). It is currently unclear whether the dual targeting of TgTPX1/2 represents bimodal targeting because of the presence of additional methionines within its N-terminal extension at positions 10 and 133. Another putative TPX gene is present in the T. gondii genome (gene model 57.m03094; TPX2 in Figure 6B), yet the profile of available expressed sequence tags suggests that this gene is only expressed in sporulated oocysts.

### Bimodal Targeting of ACN/IRP

**Figure 7:**
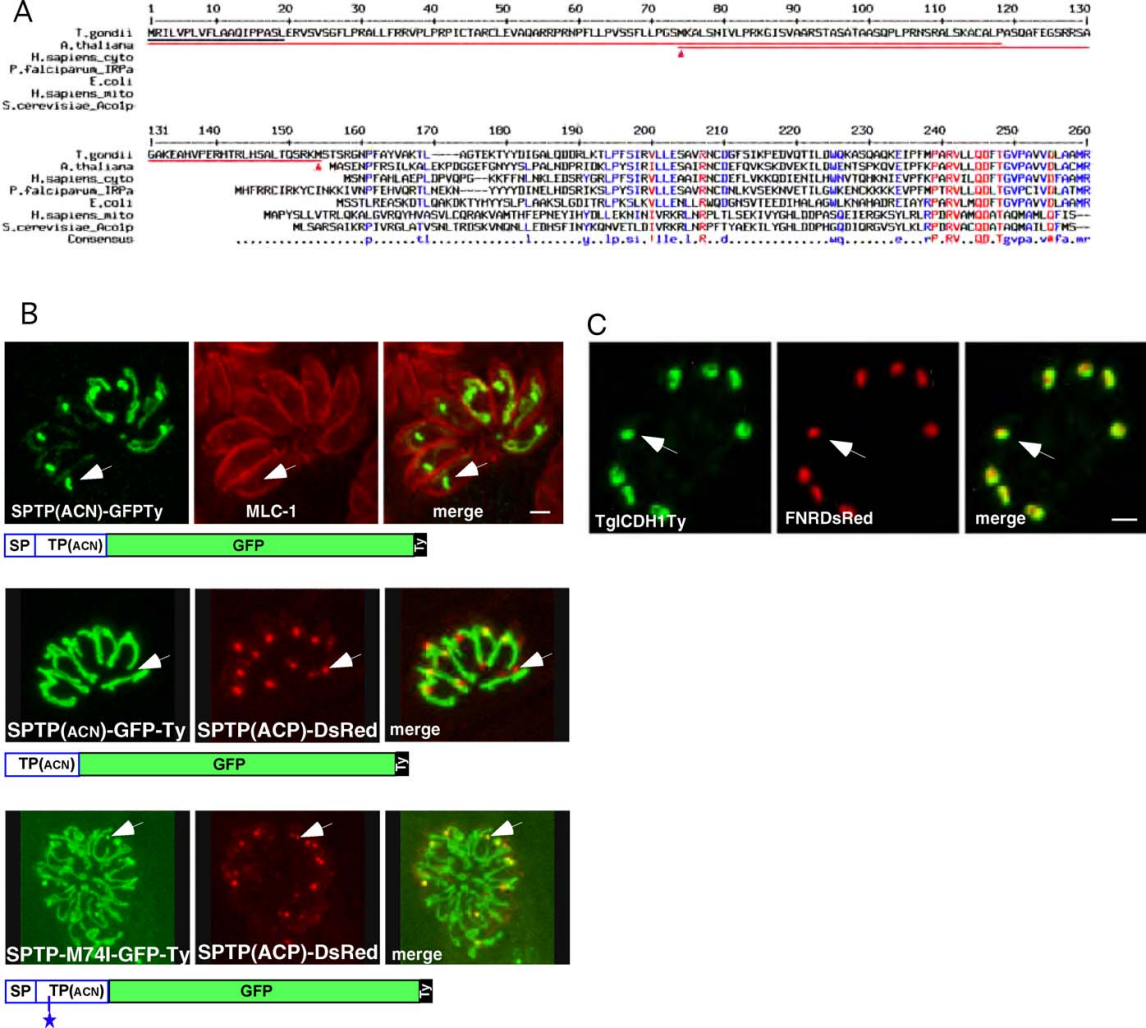
Bimodal Targeting of T. gondii ACN/IRP (A) Alignment of TgIRP/ACN with related protein sequences. Red arrowheads indicate theoretical alternative start-methionines. A putative SP as predicted by SignalP is underlined in black, and putative mitochondrial transit peptides (as predicted by TargetP or MitoProt) are underlined in red (both for the full-length sequence and for a hypothetical protein starting from the second methionine at position 74). (B) Colocalisation reveals bimodal targeting of SPTP(ACN)GFP-Ty fusion protein to the parasite mitochondrion and apicoplast. A deletion of the SP targets the fusion protein (TP(ACN)-GFPTy) to the mitochondrion only, while a mutation of the second methionine in position 74 within the TP results in bimodal targeting (SPTP(ACN)-M74I-GFP-Ty). The asterisk indicates the position of the mutation M74I. The arrows indicate the apicoplast. Scale bars represent 2 μm. (C) Colocalization of TgICDH1Ty (left panel) with the apicoplast marker FNRDsRed (middle panel) indicates a localization of ICDH1 around the periphery of the plastid organelle. Scale bars represent 2 μm.

After having identified two dually targeted antioxidant enzymes in *T. gondii,* we next aimed to determine whether proteins fulfilling other biochemical or metabolic roles might also be targeted to both mitochondrion and apicoplast. Searching for proteins expected to be present in the mitochondrion but exhibiting a bipartite targeting signal similar to those of apicoplast-targeted proteins, we identified a few candidate genes, including subunits of the pyruvate dehydrogenase (PDH) complex and the enzyme ACN/IRP. ACN is an essential enzyme of the TCA cycle in the mitochondria but may also function as an intracellular iron sensor, and in its latter role this enzyme is referred to as IRP. An IRP-like protein in *P. falciparum,* PfIRP-a has both ACN and IRP activities and was detected in the mitochondrion and the cytosol/food vacuole [41–43]. TgIRP contains a putative bipartite leader sequence for which SignalP and MitoProt predict putative signal and transit peptides of 19 and 119 amino acids in length, respectively (Figure 7A). Fusing a partial cDNA of TgIRP (the N-terminal 191 amino acids) to GFP-Ty, we created the construct SPTP(ACN)-GFP-Ty, which resulted in dual targeting of the fusion protein to both mitochondrion and apicoplast (Figure 7B). Repeating the experiment with a fusion of the full-length TgIRP cDNA with a C-terminal Ty tag confirmed dual targeting (unpublished data).

Interestingly, the apicomplexan and ciliate IRP/ACN sequences are clearly more closely related to their cytosolic counterparts of animals and plants than to the distinct mitochondrial enzymes of animals and fungi, whereas the oomycete *Phytophthora*—a distant relative of both apicomplexan parasites and ciliates—appears to contain only a typical mitochondrial ACN (Figures 7C, S1, and S2).

The N-terminal extension of TgACN contains two methionines. The second methionine at position 74 is ideal for initiating the translation of a protein strongly predicted by TargetP to be mitochondrially targeted. To test this hypothesis, we generated stable *Toxoplasma* lines expressing either TP(ACN)-GFP-Ty, starting at the second methionine in position 74, or SPTP(ACN)-M74I-GFP-Ty, with the second methionine mutated to an isoleucine (Figure S3). TP(ACN)-GFP-Ty is, as predicted by TargetP, targeted to the mitochondrion, whereas SPTP(ACN)-M74I-GFP-Ty is still bimodally targeted to the mitochondrion and the apicoplast despite the lack of potential translational initiation at position 74 (Figure 7B). This result excludes an alternative initiation of translation and is supportive of a bimodal targeting as observed for TgSOD2.

### The Two Citrate Synthases and Isocitrate Dehydrogenases of T. gondii

**Figure 8:**
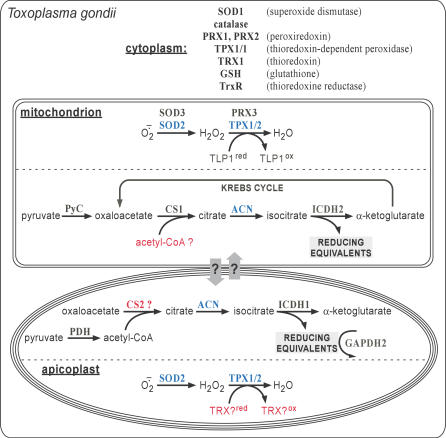
Metabolic Maps Overview of select metabolic pathways of T. gondii taken into consideration in this study. Illustrated is the established (in black or blue) or putative (in red) localization of enzymes and pathways involved in (i) antioxidant defense and (ii) in the mitochondrial Krebs cycle and in similar metabolic reactions in the apicoplast. Blue, organelle-specific enzymes and pathways; red, enzymes and reactions whose subcellular localization is hypothetical. IRE, iron-responsive element; PRX, peroxiredoxin; PyC, pyruvate carboxylase; TLP, thioredoxin-like protein.

Prompted by the unanticipated presence of T. gondii ACN in the apicoplast, we further investigated the enzymes that are—in a metabolic sense—immediately upstream and downstream of ACN in the Krebs cycle (see Figure 8), i.e., citrate synthase (CS) and isocitrate dehydrogenase (ICDH). Of the two CSs of T. gondii that could supply citrate to ACN, we demonstrate TgCS1 (59.m03414) to be mitochondrial (unpublished data), whereas the localization of the second enzyme (20.m03767/GlmHMM_0725) is currently unknown. We further showed that T. gondii possesses two ICDHs: TgICDH1 exhibits a bipartite signal and targets to the apicoplast (Figure 7C), whereas TgICDH2 carries a mitochondrial targeting signal and is located exclusively to the mitochondrion (unpublished data). Interestingly, TgICDH1 appears to localize to the periphery of the plastid organelle (Figure 7C). The presence of ICDH in the apicoplast might explain how the reducing power is produced in this organelle. Additionally, *T. gondii* possesses two genes coding for GAPDH enzymes: TgGAPDH1 is present in the cytosol, whereas TgGAPDH2 is located in the apicoplast (unpublished data) and therefore also capable of producing NADPH.

### Apicoplast Localization of the Single, Plastid-Like PDH

PDH, an enzyme complex that converts pyruvate into acetyl-CoA, commonly consists of four subunits (E1alpha, E1beta, E2, and E3), and plants and algae contain one mitochondrion-specific and one plastid-specific PDH. Apicomplexans appear to contain only a plastid-like PDH, which in P. falciparum has been reported to localize exclusively to the apicoplast using N-terminal extensions of the E1alpha, E2, and E3 subunits fused to GFP [44,45]. The lack of a mitochondrial PDH in these parasites is puzzling because it is not apparent how acetyl-CoA—the substrate of the TCA cycle—is produced in the parasite mitochondria [46,47]. Dual targeting of the PDH would have elegantly solved this metabolic puzzle; however, expression of epitope-tagged full-length cDNAs of TgPDHE1aTy and TgPDHE1bTy revealed exclusive apicoplast localization of these two enzymes by IFA (unpublished data). Recently, the absence of PDH complex in mitochondrion of T. gondii was independently confirmed [48].

## Discussion

### Dual Targeting in T. gondii

This study reports, to our knowledge for the first time, bimodal targeting to the mitochondrion and the apicoplast in an apicomplexan. The apicopast is localized within the secretory pathway, making the dual organellar targeting to both apicoplast and mitochondrion a more complex process than the dual targeting to plastid and mitochondrion in higher plants [29,49]. Targeting of TgSOD2, TgTPX1/2, and ACN/IRP is thus more similar to the cases of proteins that are dually targeted to both ER and mitochondria and plastids, such as certain cytochromes [30,31], transforming growth factor beta1 [50], the mammalian Slit3 protein [32], Alzheimer amyloid precursor protein [51], and the green algal protein disulfide isomerase RB60 [33]. In the case of TPX1/2, differential translation initiation cannot be ruled out as the underlying molecular mechanism explaining dual targeting. Production of antibodies specifically recognizing the endogenous proteins to assess dual targeting could not be achieved due to cross-reactions. TgSOD2 is 74% identical to the cytoplasmic TgSOD1, and attempts to raise antibodies against selective peptides of SOD2 failed. TgTPX1/2 is 100% identical over most of its mature protein region to the predominantly cytoplasmic TgTPX1/1. Antibodies generated against the dually targeted ACN/IRP cross-reacted with the host and gave a strong cytosolic background signal in the parasites. Nevertheless, over a dozen fusion constructs containing different protein sequences (Figure 2; Table 1) demonstrate that the plasmid and promoter (Tub8) employed in these tagging experiments are capable of targeting unambiguously proteins either to the apicoplast only (e.g., FNR-DsRed, SPTP(ACP)-GFP, TgLipA, TgICDH1, PDH subunits) or to the mitochondrion only (e.g., TgIscU, TgIscA, TgSOD3, TgPRX3, TgTLP1, TgCS1, TgICDH2). In addition, as shown in Figure 3C, stable transgenic T. gondii clones express at a comparable level the bimodally targeted TgSOD2myc to both the apicoplast and the mitochondrially targeted TgSOD2NterGFPmyc.

**Table 1:**
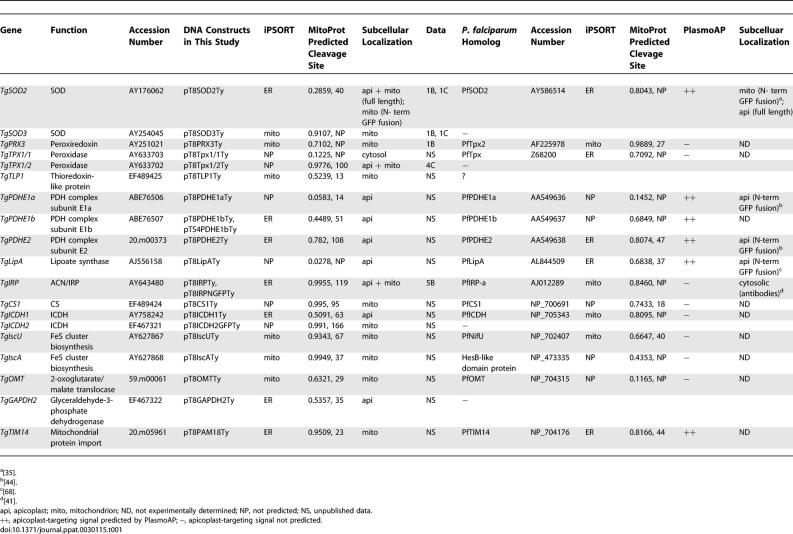
Predicted and Experimentally Determined Localization of Various Proteins Involved in Mitochondrial and/or Apicoplast Metabolism in T. gondii and P. falciparum

### A Model for Bimodal Targeting of TgSOD2 and TgACN

Analogous to models describing the bimodal targeting of other proteins [30,31,33], we suggest that the bimodal targeting of TgSOD2 and TgACN involves reduced binding of its nascent SP to the signal recognition particle, which would normally direct the protein to the ER. The apparently complete translocation of TgSOD2 into the secretory pathway and targeting to the apicoplast after the single change of an arginine to an alanine residue in its SP [16] points to a crucial role of this charged residue in preventing a very efficient interaction between the SP and the signal recognition particle. We further suggest that a certain proportion of wild-type TgSOD2 is fully translated on cytoplasmic ribosomes and binds to either cytoplasmic HSP70 or the nascent polypeptide-associated complex, which then facilitates targeting of the protein to the mitochondrion.

### Metabolic Sense of the Dual Organellar Localization of SOD2 and TPX1/2

Both mitochondria and plastids require protection from oxidative stress and damage. Even a non-photosynthetic plastid like the apicoplast is expected to produce ROS. Even for lipoic acid, a compound with potential antioxidant activity that is synthesized in the apicoplast, a significant contribution to protection against oxidative stress in this organelle has recently been called into question [52]. Our discovery of both a SOD and a thioredoxin peroxidase in the apicoplast of T. gondii thus fills this apparent metabolic gap and eliminates ROS. We also report here the presence of SOD2 in the apicoplast with no evidence of dual targeting to the mitochondrion in the intra-erythrocytic stages of P. falciparum. A study has recently established that *Plasmodium* have reduced the mitochondria with the electron transport performing just one metabolic function: the regeneration of ubiquinone [53]. This tends to suggest that a mitochondrial SOD in blood stage could be dispensable. At this point we cannot exclude that PfSOD2 also targets to the mitochondrion in other insect stages when mitochondrial functions might be more prominent.

### Metabolic Sense of the Dual Organellar Localization of ACN

T. gondii enzyme appears to be present both in the mitochondrion and the apicoplast, which—to the best of our knowledge—is the first report of an ACN/IRP localized within a plastid (although the ACN has been found associated with the etioplast fraction in plant [54]). Since the small apicoplast genome lacks readily recognizable iron-responsive elements (unpublished data), it seems likely that the T. gondii enzyme acts as an ACN in this organelle. Searching for enzymes that could deliver citrate as substrate for or make use of isocitrate resulting from the ACN reaction (see Figure 8), we localized TgCS1, one of the two citrate synthases of T. gondii (unpublished data), in the mitochondrion, whereas the subcellular localization of TgCS2 remains unknown. In contrast, we did find an ICDH, TgICDH1, present in the apicoplast (Figure 7C). This is reminiscent of the situation in plants, where an NADP-ICDH is also known to be active in the chloroplast, but here also its physiological function is not well understood [55]. As for the T. gondii apicoplast, we note that the ICDH reaction generates reducing equivalents in the form of NADPH (Figure 8), for which there is high demand in this organelle [1], and speculate that this might be the metabolic “purpose” of the dual organellar localization of ACN in T. gondii.

This study shows that dual protein targeting to the mitochondrion and apicoplast may be a widespread phenomenon in apicomplexan parasites, and that this complex targeting activity is made possible by more than one molecular mechanism. Identification of the genes that might have evolved dual targeting capability is likely to significantly change our *in silico* vision of the contents and functions of these organelles.

## Materials and Methods

### Reagents and parasite culture.

Restriction enzymes were from New England Biolabs (http://www.neb.com/), and secondary antibodies from Molecular Probes (http://probes.invitrogen.com/). T. gondii tachyzoites (RH strain wild-type and RH*hxgprt^−^*) were grown in human foreskin fibroblasts (HFF) or Vero cells in Dulbecco's Modified Eagle's Medium (DMEM) (GIBCO BRL, http://www.invitrogen.com/) supplemented with 10% fetal calf serum (FCS), 2 mM glutamine, and 25 μg/ml gentamicin. P. falciparum strain 3D7 was grown in A+ erythrocytes in RPMI-1640 medium with glutamine (Life Technologies, http://www.invitrogen.com/), 0.2% sodium bicarbonate, 25 mM HEPES, 0.2% glucose, 5% human serum, and 0.1% Albumax II (Life Technologies). Parasites were synchronized by a double sorbitol treatment as previously described [56].

### Cloning of cDNA and/or genomic locus of new T. gondii genes.

The cDNAs coding for TPX1/1, TPX1/2, ISCU, ACN (ACN/IRP), PDHE1a, PDHE1b, PDHE2, TLP1, CS1, GAPDH2, ICDH1, and ICDH2 were amplified by RT-PCR with Titan One Tube RT-PCR System (Roche Diagnostics, http://www.roche.com/) using total RNAs prepared from freshly lysed RH tachyzoites with a Triazol Kit (Invitrogen, http://www.invitrogen.com). OMT was amplified from gDNA. To define the putative translational start of TPX1/1 and TPX1/2, cDNAs were first amplified by the sense TPX1/1A and TPX1/2A and the antisense TPX1–2 primers, respectively. RT-PCR products were gel purified, cloned into pGEM T-Easy vector (Invitrogen), and sequenced. cDNAs corresponding to the open reading frame of TPX1/1 and TPX1/2 were then generated by PCR using the respective sense primers, TPX1/1B or TPX1/2B, together with the antisense TPX1–2 primers before cloning into a T. gondii expression plasmid. The genes coding for ICDH1, ISCA, TIM14, and GAPDH1 were amplified by PCR using genomic DNA from T. gondii strain RH. All oligonucleotide primers used for PCRs are listed in Table 2.

**Table 2:**
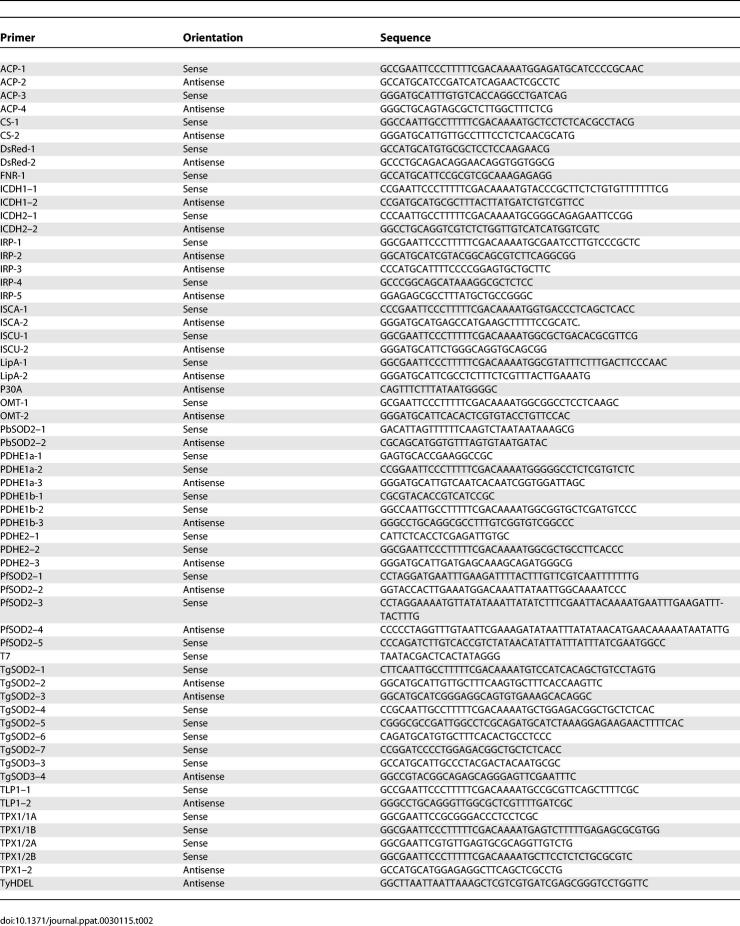
Oligonucleotide Primers Used in This Study

### Construction of T. gondii expression plasmids.

The cloning of plasmids pT8SOD2Ty, pT8SOD3Ty, and pT8PRX3Ty was described previously [17]. Apart from FNR-DsRed, which is a generous gift from Boris Striepen [57] (University of Georgia, United States), all T. gondii expression plasmids were modified from the original vectors pTUB8MycGFPPf.myotailTy-1-HX [58]. The cDNAs of TPX1/1, TPX1/2, ISCU, IRP, PDHE1a, PDHE1b, PDHE2, TLP1, CS1, OMT, GAPDH2, and ICDH2, and the genomic DNAs of ICDH1, ISCA, and Tim14 were cloned into pTUB8MycGFPPf.myotailTy-1-HX to generate TPX1/1Ty, TPX1/2Ty, ISCUTy, ACNTy, PDHE1aTy, PDHE1bTy, PDHE2Ty, TLP1Ty, CS1Ty, OMTTy, ICDH2Ty, ICDH1Ty, ISCA Ty, GAPDH2Ty, and PAM18Ty. The C-terminal Ty-1 tag of SOD2Ty and pTUB8MycGFPPf.myotailTy-1-HX was changed to a myc tag to generate SOD2myc and pT8mycGFPmycHX. The PCR fragment SOD2 SPTP (aa 1–91) was generated with primers SOD2–1 and SOD2–3 using plasmid template SOD2Ty and was cloned between EcoRI and NsiI of pT8mycGFPmycHX to generate SPTP-GFPmyc. SPTP-DsRed was generated by replacing the GFPmyc in SPTP-GFPmyc with DsRed between NsiI and PacI restriction enzyme sites. The DsRed fragment was amplified from the plasmid template pDsRed1–1 (Clontech, http://www.clontech.com/) using the primers DsRed-1 and DsRed-2. SPTP-SOD3Ty was generated by replacing the GFPmyc of SPTP-GFPmyc with the SOD3 mature protein (aa 57–258) fused with a Ty-1 tag amplified by PCR using the primers SOD3–2 and SOD3–3, and the plasmid template SOD3Ty. The transit peptide of SOD3 (aa 1–56), together with the promoter region from the plasmid SOD3Ty, was amplified by PCR primers, T7 and SOD3–4, and was cloned between KpnI and BsiWI in the plasmid SOD2Ty to create TP(SOD3)-SOD2Ty. The deletion construct ΔTPSOD2Ty was cloned in a two-step strategy. A KasI- and NsiI-flanked GFP PCR product amplified with the primers SOD2–5 and p30A using template plasmid SPTP-GFPTy was first used to replace the transit peptide and mature SOD2 protein in plasmid SOD2Ty and resulted in SPGFPTy. GFP was then replaced with a fragment of mature SOD2 protein flanked with Nsi sites (amplified by PCR with primers SOD2–6 and SOD2–2) to form SP-SOD2Ty. For SP-SOD2TyHDEL, a PCR fragment was generated using primers SOD2–1 and TyHDEL, which contains the coding sequence of a C-terminal ER retention signal, using template plasmid ΔTPSOD2Ty. The resulting PCR product was then cloned between the EcoRI and PacI sites of pTUB8MycGFPPf.myotailTy-1-HX. To generate ΔSPSOD2Ty, a truncated SOD2 fragment (aa 26–287) was amplified by PCR using primers SOD2–4 and SOD2–2 from plasmid template SOD2Ty, and then cloned between the EcoRI and PacI sites of pTUB8MycGFPPf.myotailTy-1-HX. The apicoplast marker construct SPTP(ACP)-GFP was created by exchanging the SPTP of SOD2 in SPTP-GFPTy with the N-terminus of ACP (aa 1–102) amplified by PCR with primers ACP-1 and ACP-2 using genomic DNA template. To generate SP-FNR-DsRed and SP-ACPTy, PCRs were performed with the primer pairs FNR-1 and DsRed-2, and ACP-3 and ACP-4 using the plasmid FNR-DsRed and T. gondii genomic DNA as templates, respectively. The resulting PCR fragments were cloned between the NsiI restriction enzyme sites in SP-SOD2Ty. The plasmid SPTP(ACN)-GFP-Ty was constructed by replacing the SPTP of SPTPGFPTy with the partial cDNA of ACN/IRP (aa 1–191). The expression vector SPTP(ACP)DsRed was constructed by replacing between EcoRI to Nsil of the original vector, TgSPTP-DsRed, with the correspondingly enzyme-restricted genomic DNA fragment coding for ACP (aa 1–102), which was amplified with PCR primers ACP-1 and ACP-2. Site-directed mutagenesis to generate SPTP(ACN)-M74I-GFPTy, where Met74 is mutated to IIe, was performed with primers IRP-4 and IRP-5, using the QuickChange II site-directed mutagenesis kit (Stratagene, http://www.stratagene.com/), according to the manufacturer's instructions.

### Cloning of *Plasmodium* SOD2.

The N-terminus of the SOD2 gene was cloned from a P. berghei cDNA library by PCR using primers PbSOD2–1 and PbSOD2–2. The cDNA library was kindly generated and provided by J. T. Dessens (Imperial College London, United Kindgom). The full-length SOD2 gene was cloned from P. falciparum. Total RNA was isolated from parasites using Trizol (Invitrogen). Reverse transcriptase (RT) reaction on 2 μg of total RNA was performed with Superscript II (GIBCO BRL), and PCR reactions with LA Taq DNA polymerase (Takara, http://www.takara-bio.com/). Primers PfSOD2–1 and PfSOD2–2 were designed to amplify the gene starting with the “second” methionine (see Figure 2), i.e., as predicted by the PHAT3 gene prediction program [35] (accession AAT11554) and as annotated (PFF1130c) in PlasmoDB (http://www.plasmodb.org/). A second sense primer, PfSOD2–3, was used to amplify the gene starting with the “first” methionine 11 amino acids upstream of the start of the PHAT3-predicted gene (see Figure 2). Amplification reactions were performed as follows: one cycle at 94 °C for 2 min; 35 cycles (each) at 94 °C for 30s, 50 °C for 30s, 68 °C for 90s; and one cycle at 68 °C for 10 min. Subcellular targeting of PfSOD2 in P. falciparum was analyzed by fusing the full-length PfSOD2 sequence (see Figure 1A) to GFP in the pARL-GFP plasmid (kind gift from Brendan Crabb and Alan Cowman, WEHI, Melbourne, Australia). The *CRT* promoter in pARL-SOD2-GPF was replaced by a genomic region corresponding to the promoter and 5′UTR of *PfSOD2,* 1,450 bps upstream of the first ATG of *PfSOD2* with the primers PfSOD2–4 and PfSOD2–5.

### Parasite transfection and selection of clonal stable lines.

Transient transfections of T. gondii strain RH *hxgprt^−^* were undertaken as previously described [59]. Stable transformants were selected for expression of hypoxanthine-xanthine-guanine-phosphoribosyltransferase (HXGPRT) [60]. P. falciparum erythrocytic stage parasites were transfected as previously described [61]. Transfection of *Plasmodium* clones 3D7 and D10 were carried out by electroporation and drug selection using 5 nM of WR99210 for the human dhfr-based plasmid pARL1 as previously described [62].

### IFA and confocal microscopy.

Intracellular parasites grown in HFF on glass slides were fixed with 4% paraformaldehyde with or without 0.05% glutaraldehyde for 20 min. Following fixation, slides were rinsed in PBS-0.1M glycine. Cells were then permeabilized in PBS-0.2% Triton-X100 for 20 min and blocked in the same buffer with 2% FCS. Slides were incubated for 60 min with primary antibodies diluted in PBS-1% FCS, washed, and incubated for 60 min with Alexa488- or FITC-coupled goat anti-mouse IgGs diluted in PBS-1% FCS. Slides were mounted in Fluoromount G (SouthernBiotech, http://www.southernbiotech.com/). Confocal images were collected with a Leica (http://www.leica.com/) laser scanning confocal microscope (TCS-NT DM/IRB and SP2) using a 100× Plan-Apo objective with NA 1.4. Single optical sections were recorded with an optimal pinhole of 1.0 (according to Leica instructions) and 16 times averaging. Stacks of sections were recorded at ∼0.2-μm vertical steps and projected using the maximum projection tool. All other micrographs were obtained with a Zeiss Axiophot (http://www.zeiss.com/) with a camera (Photometrics Type CH-250; http://www.photomet.com/). Adobe Photoshop (Adobe Systems, http://www.adobe.com/) was used for image processing. Green fluorescence of GFP-expressing P. falciparum parasites was observed and captured in live cells by confocal microscopy. Active mitochondria were stained with the fluorescent dye MitoTracker (Molecular Probes).

### Electron microscopy and immunogold-labelling.

Freshly lysed parasites were fixed in 0.1 M cacodylate buffer (pH 7.2) containing 2% formaldehyde, 2% glutaraldehyde, 5 mM calcium chloride, and 5% sucrose for 2 h at room temperature. Fixed cells were dehydrated in a graded ethanol series and embedded in LR gold resin (Agar Scientific, http://www.agarscientific.com/). Ultrathin serial sections were incubated with anti-Ty-1 primary antibody, followed by anti-mouse IgG antibodies coupled to 18-nm gold particles (Jackson ImmunoResearch, http://www.jacksonimmuno.com/). Finally, sections were stained with uranyl acetate and lead citrate and observed using a Philips CM100 transmission electron microscope (http://www.philips.com/).

### Bioinformatics.

Putative SPs were predicted using SignalP and iPSORT [63–65], and putative mitochondrial transit peptides using TargetP [66] and MitoProt II [67] (http://ihg.gsf.de/ihg/mitoprot.html). Multiple protein sequence alignments were generated with ClustalX 1.83 using the following “Fast-Approximate” pairwise alignment parameters: gap penalty = 4, K-tuple size = 1, top diagonals = 50, window size = 50. Alignment positions containing gaps in >50% of the sequences were excluded from phylogenetic analyses. Database accession numbers are listed in the Accession Numbers section. Phylogenetic analyses were carried out using ClustalX 1.83 with the “Correct for Multiple Substitutions” option enabled, and bootstrapping was performed by running 500 replicates.

## Supporting Information

Figure S1 Phylogenetic Tree of ACN/IRP Protein Sequences Based on Neighbor-Joining Bootstrap Analysis (ClustalX 1.83, 500 Replicates) This is a fully annotated version of the phylogenetic tree shown in Figure 7C.
(348 KB PDF)

Figure S2 Unrooted Phylogenetic Tree of ACN/IRP Protein Sequences Based on Neighbor-Joining Bootstrap Analysis (500 Replicates) Bootstrap values for select branches are indicated in the tree. See Figure S1 for a fully annotated version of this tree. Dd, *Dictyostelium discoideum;* Pc, *Plasmodium chabaudi;* Pf, *Plasmodium falciparum;* Pr, *Phytophthora ramorum;* Ps, *Phytophthora sojae;* Py, *Plasmodium yoelii;* Ta, *Theileria annulata;* Tg, *Toxoplasma gondii;* Tp, *Theileria parva;* Tt, Tetrahymena thermophila.
(305 KB PDF)

Figure S3 Overview of Recombinant Constructs Expressed in T. gondii
Various T. gondii full-length proteins and protein domains were expressed as tagged fusion proteins (employing Ty-protein tag and GFP) to determine their intracellular localization. The overview shows the name of the recombinant construct, the experimentally determined localization, and a diagrammatic representation of the fusion protein. In the fusion SPTP(ACN)M74I-GFPTy, the second methionine within the N-terminal extension has been mutated to an isoleucine residue.
(49 KB PDF)

### Accession Numbers

The accession and ID numbers of genes and proteins discussed in the text are A. thaliana GPX1 (NP_180080); A. thaliana IRP (NP_195308); AthGPX2 (NP_180715); AthGPX3 (NP_181863); Bradyrhizobium sp. GPX (ZP_00862752); Escherichia coli IRP (CAA42834); HesB-like domain protein (NP_473335); Homo sapiens cytosolic IRP (CAH72598); H. sapiens mitochondrial IRP (AAB38416); PbSOD2 (EF455011); PfCS1 (NP_700691); PfICDH (NP_705343); PfIRP-a (AJ012289); PfLipA (AL844509); PfNifU (NP_702407); PfOMT (NP_704315); PfPDHE1a (AAS49636); PfPDHE1b (AAS49637); PfPDHE2 (AAS49638); PfSOD2 (AY586514); PfTIM14 (NP_704176); PfTPRX2 (AF225978); PfTPX (Z68200); Rickettsia prowazekii SOD (Q9ZD15); Saccharomyces cerevisiae IRP (NP_013407); Synechocystis sp. SOD(S75466); Synechocystis sp. GPX (BAA18344); TgCS1 (EF489424); TgCS1, citrate synthase (EF489424); TgGAPDH2 (892132); TgGAPDH2, glyceraldehyde-3-phosphate dehydrogenase (EF467322); TgICDH1 (AY758242); TgICDH1, isocitrate dehydrogenase (AY758242); TgICDH2 (892126); TgICDH2, isocitrate dehydrogenase (EF467321); TgIRP/ACN (AAT68238); TgIRP, aconitase/iron regulatory protein (AY643480); TgIscA, FeS cluster biosynthesis (AY627868); TgIscU, FeS cluster biosynthesis (AY627867); TgLipA, lipoate synthase (AJ556158); TgOMT, 2-oxoglutarate/malate translocase (59.m00061); TgPDHE1a, PDH complex subunit E1a (ABE76506); TgPDHE1b, PDH complex subunit E1b (ABE76507); TgPDHE2, PDH complex subunit E2, (20.m00373); TgPRX3, peroxiredoxin (AY251021); TgSOD1 (AAC63943); TgSOD2, superoxide dismutase (AY176062); TgSOD3 (AY254045); TgTIM14, mitochondrial protein import (20.m05961); TgTLP1 (EF489425); TgTLP1, thioredoxin-like protein (EF489425); TgTPX1 (AY043228); TgTPX2 (BM039715); TgTPX1/1, peroxidase (AY633703); and TgTPX1/2 (AY633702).

Sequences were obtained from the GenBank database (http://www.ncbi.nlm.nih.gov/Genbank/index.html) and from the following sources: ToxoDB (http://www.ToxoDB.org/) and PlasmoDB (http://www.plasmodb.org/).

## Abbreviations

ACN: aconitase
ACP: acyl carrier protein
CS: citrate synthase
ER: endoplasmic reticulum
FNR: ferredoxin NADP+ reductase
GAPDH: glyceraldehyde-3-phosphate dehydrogenase
GFP: green fluorescent protein
ICDH: isocitrate dehydrogenase
IFA: immunofluorescence assay
IRP: iron regulatory protein
Pb: Plasmodium berghei
PDH: pyruvate dehydrogenase
Pf: Plasmodium falciparum
ROS: reactive oxygen species
RT-PCR: reverse transcription PCR
SOD: superoxide dismutase
SP: signal peptide
TCA: tricarboxylic acid
Tg: Toxoplasma gondii
TPX: thioredoxin-dependent peroxidase
