# Supplementary figures and images for: Dual Targeting of Antioxidant and Metabolic Enzymes to the Mitochondrion and the Apicoplast of Toxoplasma gondii
Source: PLoS Pathog. 2007 Aug 31;3(8):e115. doi: 10.1371/journal.ppat.0030115 (PMC1959373; doi:10.1371/journal.ppat.0030115)

Supplementary Figure S1

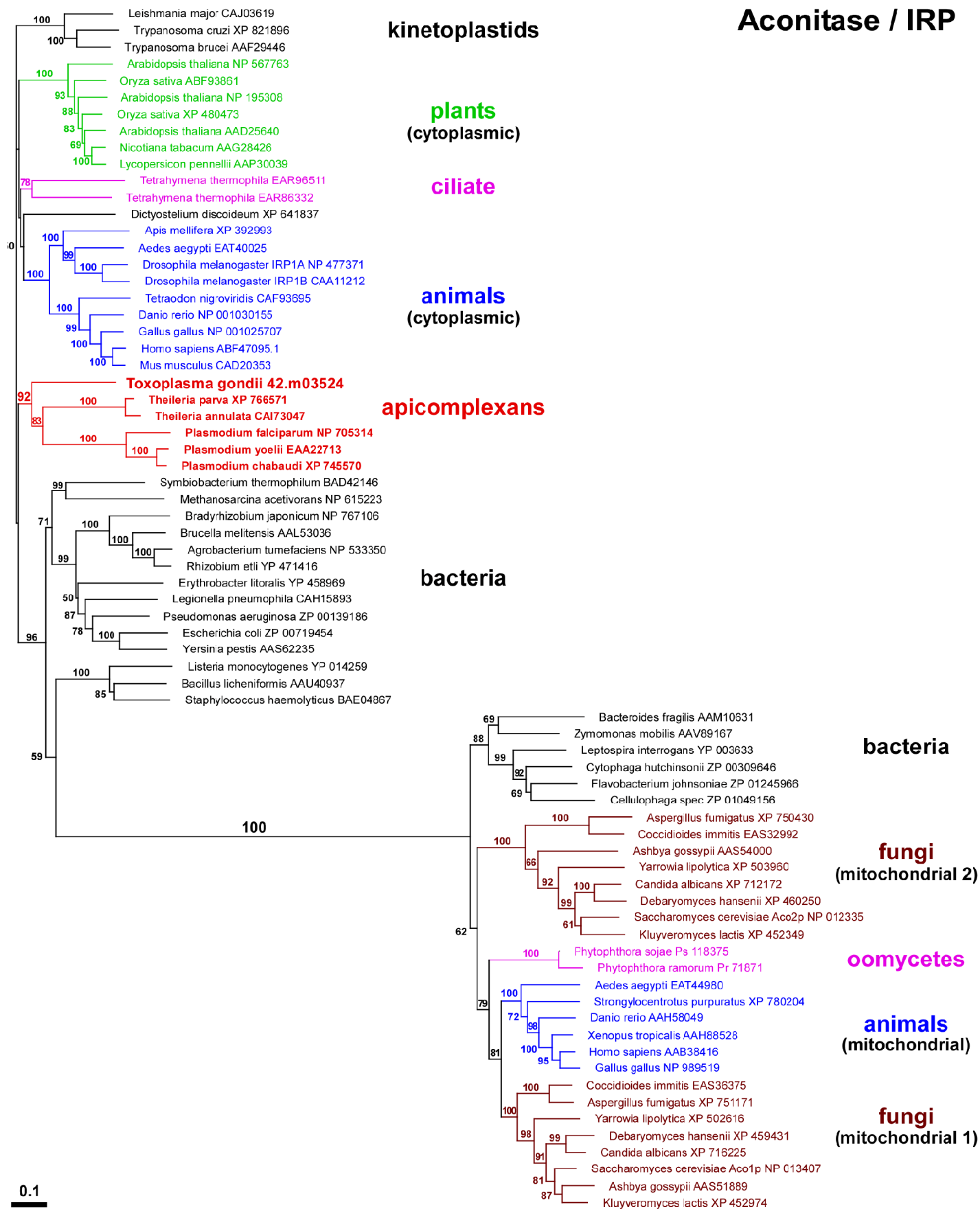

Supplement: Figure S1 — This is a fully annotated version of the phylogenetic tree shown in Figure 7C. (348 KB PDF) [file ppat.0030115.sg001.pdf]

Supplementary figure S2

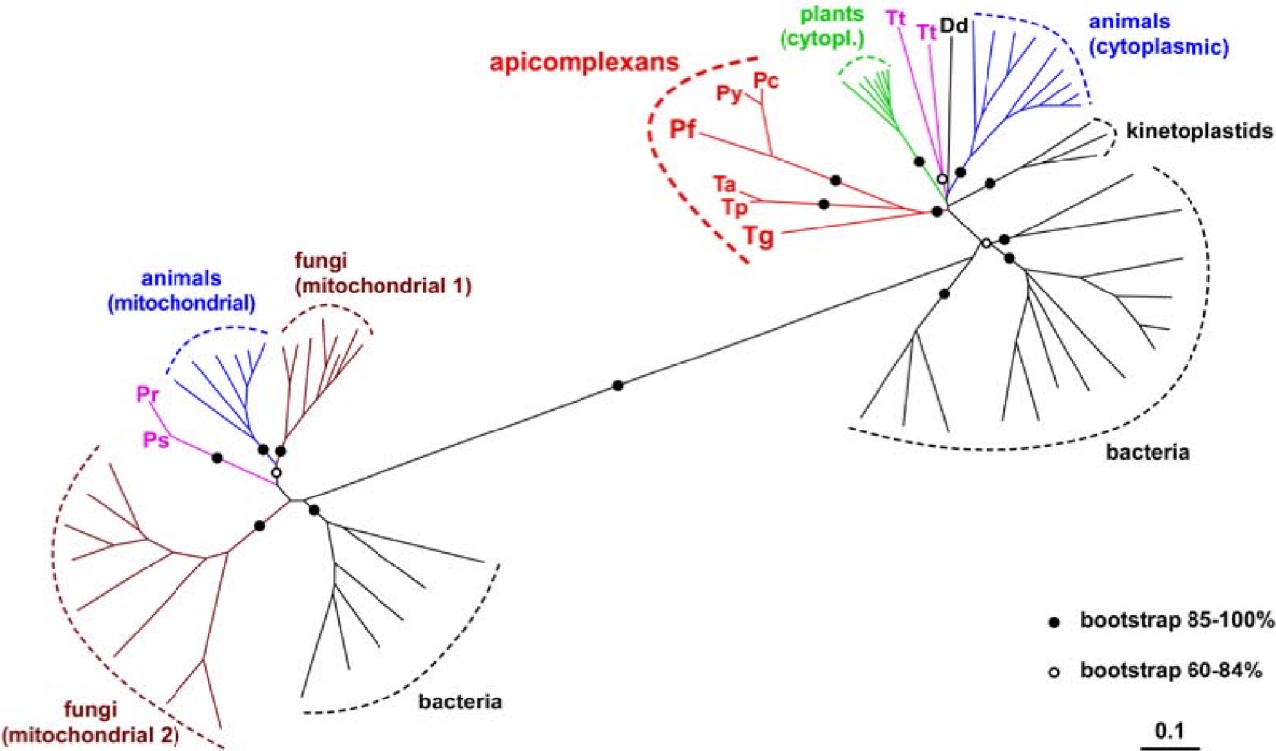

Supplement: Figure S2 — Bootstrap values for select branches are indicated in the tree. See Figure S1 for a fully annotated version of this tree. Dd, Dictyostelium discoideum; Pc, Plasmodium chabaudi; Pf, Plasmodium falciparum; Pr, Phytophthora ramorum; Ps, Phytophthora sojae; Py, Plasmodium yoelii; Ta, Theileria annulata; Tg, Toxoplasma gondii; Tp, Theileria parva; Tt, Tetrahymena thermophila. (305 KB PDF) [file ppat.0030115.sg002.pdf]
