# Supplementary material for: Dual Targeting of Antioxidant and Metabolic Enzymes to the Mitochondrion and the Apicoplast of Toxoplasma gondii
Source: PLoS Pathog. 2007 Aug 31;3(8):e115. doi: 10.1371/journal.ppat.0030115 (PMC1959373; doi:10.1371/journal.ppat.0030115)

Supplementary figure S3

Construct	Localization
**TPX1/1Ty**	Cytosol
**TPX1/2Ty**	mitochondrion & apicoplast
**SPTP(ACN)-GFP-Ty**	mitochondrion & apicoplast
**TPACN-GFP-Ty**	mitochondrion
**SPTP(ACN)-M74IG-FP-Ty**	mitochondrion & apicoplast

Schematic of recombinant protein

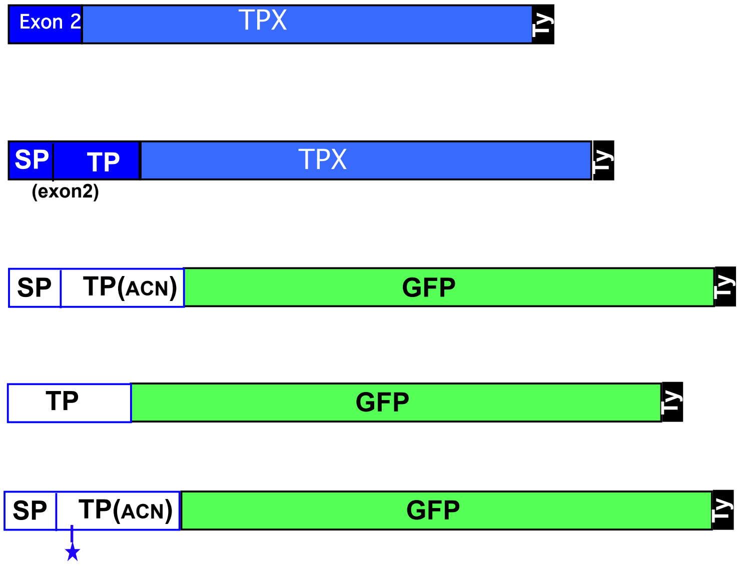

Supplement: Figure S3 — Various T. gondii full-length proteins and protein domains were expressed as tagged fusion proteins (employing Ty-protein tag and GFP) to determine their intracellular localization. The overview shows the name of the recombinant construct, the experimentally determined localization, and a diagrammatic representation of the fusion protein. In the fusion SPTP(ACN)M74I-GFPTy, the second methionine within the N-terminal extension has been mutated to an isoleucine residue. (49 KB PDF) [file ppat.0030115.sg003.pdf]
